# How Well Can We Extract the Permanent Displacement from Low-Cost MEMS Accelerometers?

**DOI:** 10.3390/s17112643

**Published:** 2017-11-16

**Authors:** Jyh Cherng Jan, Wei-An Chao, Yih-Min Wu, Chien-Chih Chen, Cheng-Horng Lin

**Affiliations:** 1 Department of Earth Sciences, National Central University, Jhongli 320, Taiwan; jyhcherngjan@gmail.com (J.C.J.); chienchih.chen@g.ncu.edu.tw (C.-C.C.); 2 Institute of Earth Sciences, Academia Sinica, Taipei 11529, Taiwan; drymwu@ntu.edu.tw (Y.-M.W.); lin@earth.sinica.edu.tw (C.-H.L.); 3 Department of Civil Engineering, National Chiao Tung University, Hsinchu 30010, Taiwan; 4 Department of Geosciences, National Taiwan University, Taipei 10617, Taiwan

**Keywords:** low-cost MEMS accelerometers, earthquake permanent displacement, seismic hazard mitigation, *P*-Alert

## Abstract

Following the recent establishment of a high-density seismic network equipped with low-cost micro-electro-mechanical system (MEMS) *P*-wave-alert-device (*P*-Alert) by the earthquake early warning (EEW) research group at the National Taiwan University, a large quantity of strong-motion records from moderate-magnitude earthquakes (M_L_ > 6) around Taiwan has been accumulated. Using a data preprocessing scheme to recover the dynamic average embedded within the *P*-Alert data, we adopted an automatic baseline correction approach for the *P*-Alert accelerograms to determine the coseismic deformation (Cd). Comparisons between the Cd values determined using global positioning system (GPS) data, strong-motion records from the *P*-Alert network, and data from the Taiwan Strong Motion Instrumentation Program (TSMIP) demonstrates that the near-real-time determination of Cd values (>2 cm), which provide crucial information for seismic hazard mitigation, is possible using records from low-cost MEMS accelerometers.

## 1. Introduction

**Figure 1:**
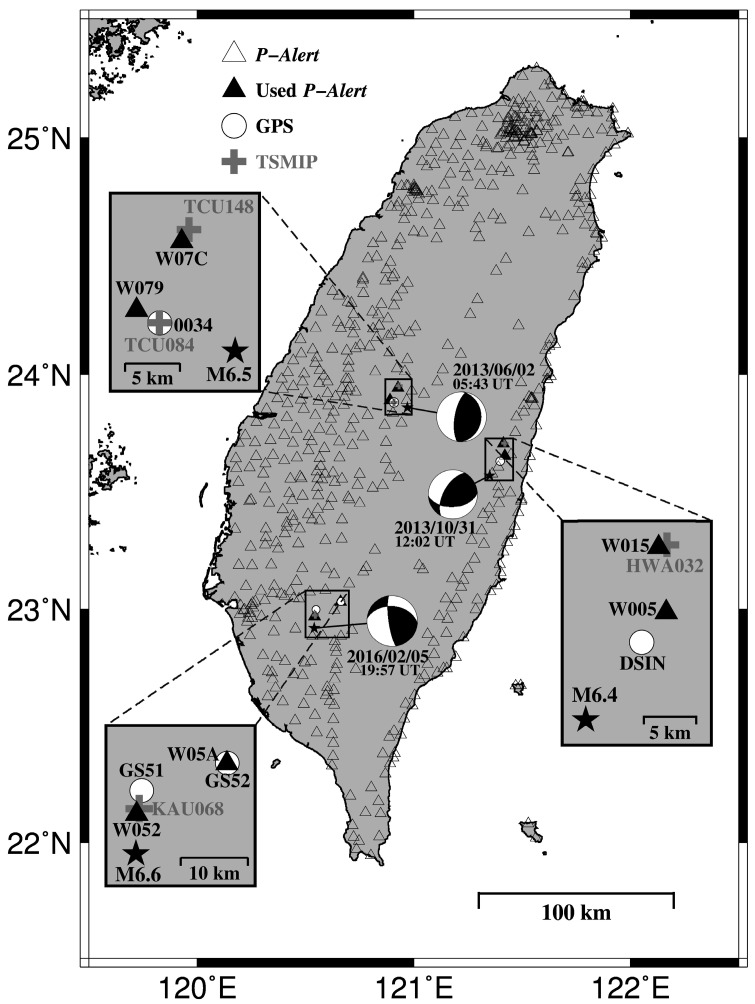
Distribution of *P*-Alert stations shown by open triangles. Black stars indicate the locations of three earthquakes plotted with the focal mechanism solutions from CWB. Solid triangles, white circles, and crossed symbols represent stations of the *P*-Alert, the GPS and the TSMIP used in this study, respectively.

Over the past few decades, numerous studies on the determination of coseismic deformation (Cd) using strong-motion data have been published (e.g., [1,2,3,4,5,6]). In Taiwan, the Central Weather Bureau (CWB) implemented the Taiwan Strong Motion Instrumentation Program (TSMIP) to provide high-quality accelerograms for moderate-to-large sized earthquakes. More than 680 free-field seismic stations are spread out over inland Taiwan, but only approximately 109 stations provide real-time data transmission. This station coverage is insufficient for the rapid and accurate reporting of both earthquake parameters (e.g., location and magnitude) and near-real-time shaking maps. To improve this lack of real-time streaming data, an additional dense seismic network is needed. During recent years, the earthquake early warning (EEW) research group at the National Taiwan University deployed a dense seismic network based on micro-electro-mechanical system (MEMS) [7] accelerometers (dubbed *P*-wave-alert-device (*P*-Alert) [8]). Figure 1 shows the station distribution of the *P*-Alert network, with a total of 607 stations as of July 2016. These stations, installed on the wall of buildings, have facilitated seismological studies throughout Taiwan, particularly for seismic hazard assessment purposes, including regional [9,10] and on-site [11] EEW systems, near-real-time shaking maps [12,13], and structural health monitoring [14]. Most of the stations in the current *P*-Alert network were installed on the 1st (76 percent of total station number) and 2nd (19 percent) floors of buildings, and only a few *P*-Alert stations were put in basements or on the 3rd floor (or higher). Most schools in Taiwan are one- or two-story buildings. 

The main goal of this study is the use of *P*-Alert accelerograms to determine real-time permanent displacements induced by earthquakes. Another possible solution is to extract Cd estimates from geodetic continuous global positioning system (CGPS) instruments [15] maintained by the Institute of Earth Sciences, Academia Sinica (IESAS), the Ministry of Economic Affairs (MEA) in Taiwan, and the CWB. However, geodetic data are not available for the real-time estimation of Cd values due to the necessity of longer time series of data for regression analysis. In this study, we collect strong-motion records from the *P*-Alert and the TSMIP seismic networks for moderate-magnitude (M_L_ > 6) inland earthquakes during a monitoring period of 2013–2016. After adopting an algorithm for baseline corrections proposed by [6], a further comparison of the Cd results obtained from accelerograms with GPS-based estimations [16,17,18] is able to reveal the feasibility of the Cd determination using real-time *P*-Alert data.

## 2. Data and Method 

Each station in the TSMIP is equipped with a force-balance accelerometer with a sampling rate of 200 Hz (or higher). The signal resolution of a MEMS *P*-Alert accelerometer is 16 bits with a dynamic range of ±2 g, and the sampling rate is 100 samples per second. The *P*-Alert device is small in size, with dimensions of 125 × 105 × 30 mm^3^, a weight of 450 g and a power consumption of 3.5 W. The *P*-Alert works with a frequency response of 0.05–20 Hz and an operating temperature of −10 to 60 °C. Here, a low-pass filter with a corner frequency of 10 Hz is applied to the real-time signal to avoid contamination from ambient noise. The timing control is based on Internet Network Time Protocol (NTP) synchronization. The self-noise level of *P*-Alert is about 0.1 gal (1 gal = 1 cm/s^2^), which is higher compared with the traditional accelerometers, but its price is ten times cheaper. The above-mentioned advantages facilitate the widely spaced deployment of the *P*-Alert stations around Taiwan island, advancing the studies of the real-time seismology, especially for the EEW [9,10,11]. 

During the preprocessing of *P*-Alert data records, the *P*-Alert recorded signals (P_record_) are computed from the dynamic averaging of the original signals (P_original_) at each time step (0.01 s). The formula used within the dynamic averaging scheme for recorded signals can be expressed as follows:

P_record_(i) = P_original_(i) − D(i) (1)

D(i) = 0.001 × P_original_(i) + 0.999 × D(i − 1) (2)

where D is the dynamic average and i is the i-th time step. Based on Equations (1) and (2), we can calculate P_original_ for the input data within the Cd determination. Here, signal recovery processing is conducted for the three-component accelerograms.

**Figure 2:**
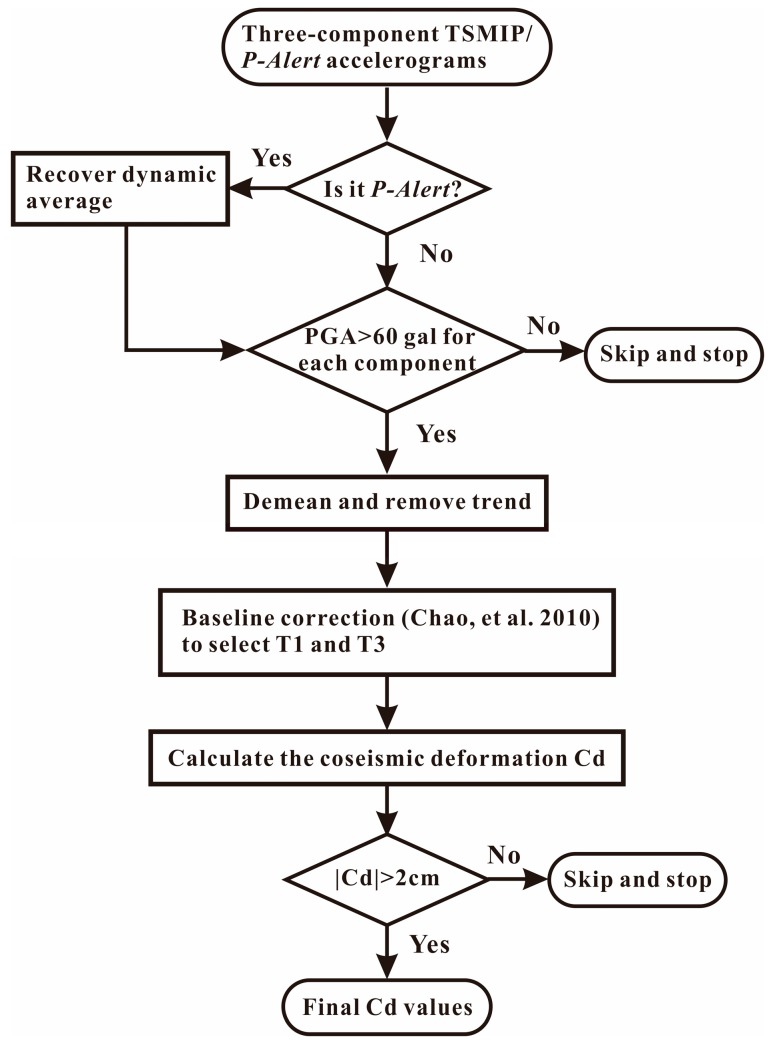
The flowchart showing the procedure of Cd determination in this study.

In consideration of three moderate-magnitude (M_L_ > 6; stars shown in Figure 1) earthquakes, we used strong-motion records with observed peak ground acceleration (PGA) values larger than 60 gal [5] from the *P*-Alert and TSMIP networks. Then, we removed the mean and linear trends prior to the *P*-wave arrival and determined the Cd values using the automated baseline correction approach developed by [6], which can automatically select the crucial time points (see T1 and T3 plotted in Figure 1 of [6]) for the baseline correction based on the seismic energy ratio distribution. In general, T1 is the starting time at which the ground begins to shake, and T3 is the point in time at which the ground has reached a permanent displacement. The flowchart utilized to compute the Cd is shown in Figure 2. To compare the results derived from the strong-motion records, we also collected the Cd measurements [16,17,18] derived from GPS stations located around the *P*-Alert/TSMIP stations.

## 3. Results

**Figure 3:**
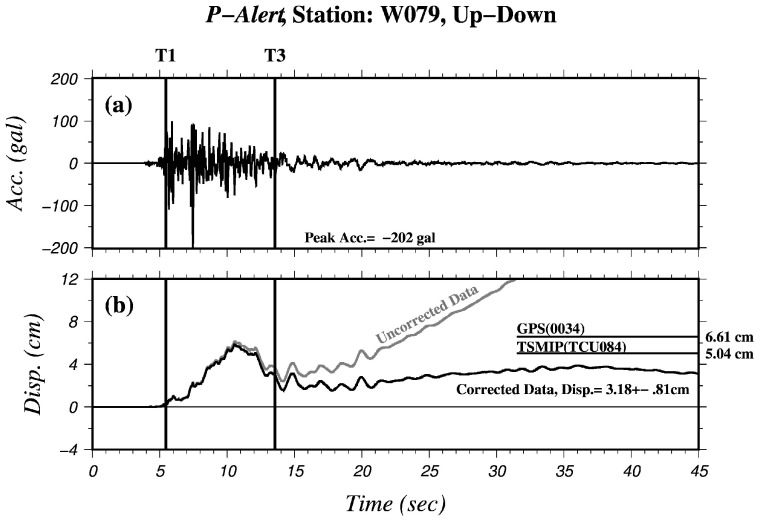
Vertical component time series of (**a**) accelerogram and (**b**) uncorrected (grey) and corrected (black) displacements recorded at *P*-Alert station W079 for the 2 June 2013 earthquake. Two horizontal lines depict the Cd results of the GPS and the TSMIP. Vertical lines mark time points of T1 and T3 for baseline correction.

**Figure 4:**
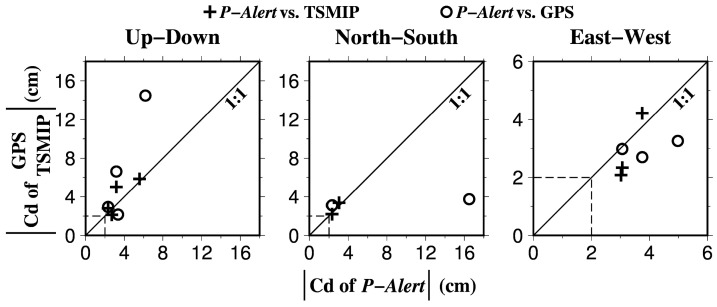
Comparison of three-component absolute Cd values computed from the strong-motion records (TSMIP and *P*-Alert) and the geodetic data (GPS). Dashed line indicates a threshold of Cd value of 2 cm.

The *P*-Alert vertical accelerograms, original displacement, and corrected displacement recorded at station W079 for the 2 June 2013 Nantou earthquake are shown in Figure 3. In the case displayed in Figure 3b, the Cd value computed from the *P*-Alert data is 3.18 cm with a standard deviation (SDV) of 0.81 cm, which is smaller than the results obtained using the GPS (0034: 6.61 cm) and the TSMIP (TCU084: 5.04 cm) records. These discrepancies might have been caused by the different locations of the stations, as the GPS and TSMIP stations were much closer to the epicenter of the earthquake (Figure 1). Table 1 gives a summary of the three-component Cd values estimated from the *P*-Alert, GPS and TSMIP data records, and Figure 4 shows a comparison of all of the Cd results. Overall, the Cd values acquired from the *P*-Alert network are roughly consistent with the results retrieved from the GPS and/or TSMIP records when the stations were nearly co-located with the *P*-Alert stations. For example, a comparable result between the *P*-Alert (W07C) and TSMIP (TCU148) stations is shown in Table 1. In contrast, there is an obvious discrepancy in the Cd estimations between the *P*-Alert (W005) and GPS (DSIN) stations due to the large distance (~4 km) between the station locations (Figure 1 and Table 1).

## 4. Discussion and Conclusions

After using an available data preprocessing scheme to compute the original *P*-Alert records, we are able to extract the permanent displacement based on the *P*-Alert data using the automatic baseline correction approach developed by [6]. Systematic comparisons between the results acquired from the *P*-Alert, GPS and TSMIP stations demonstrate that near-real-time Cd values can be successfully determined, especially for cases with PGA values larger than 60 gal and Cd values greater than 2 cm (Figure 4). 

**Figure 5:**
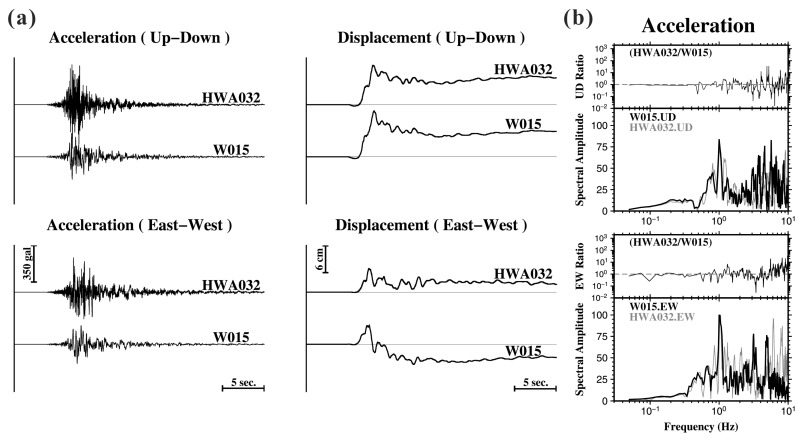
(**a**) Comparison of accelerograms (left) and corrected displacements (right) in the vertical and horizontal (East-West) components recorded at the TSMIP (HWA032) and *P*-Alert (W015) stations. (**b**) Fourier spectral amplitude of acceleration records and amplitude ratio between the W015 and HWA032 stations for the vertical (upper) and East-West (bottom) components. A dashed line is shown to indicate a ratio value of 1.

In general, buildings have a natural oscillation period that corresponds to building height. The natural frequencies of 1 to 20 story normal reinforced concrete and steel buildings are in the range of 0.5–20 Hz. Ground vibrations will be amplified if their period matches the natural resonance of a building. The stations used in this study are located at 1st or 2nd floors of buildings (Table 1). Thus, the filtration of building effects may not have directly contaminated the computed Cd values, since the permanent displacement is generally recorded within relatively long-period portions of the accelerograms. However, the low-frequency ground shaking could still be amplified by the site condition of soft sediments. Moreover, building effects also depend on structural characteristics such as the stiffness, strength and ductility of a building. These effects on the high-frequency portion (e.g., peak ground acceleration, PGA) have been systematically investigated in our research group [19]. Previous studies [20,21] have illustrated the ground tilt effects based on different tilt sensitivities in the horizontal and vertical components. In practice, buildings are much stiffer in the vertical direction. Thus, we consider that the back-and-forth vibration of a building is likely to influence the *P*-Alert records, especially for the horizontal component. In the above case, we would expect to see relatively larger discrepancies in the comparative investigations of horizontal Cd values. However, when the *P*-Alert stations are close to the TSMIP stations, the Cd values extracted from the *P*-Alert stations are consistent with the results of the TSMIP stations (Table 1). The only exception is the W015-HWA032 station pair (inter-station distance ~600 m), which shows an obvious difference (different polarity) in the east-west components, while a consistent pattern in their vertical components is observed. We carefully examined the time series of the acceleration and the corrected displacements (Figure 5a) and applied spectral analyses (Figure 5b). The ratio of spectral amplitudes reveals the existence of discrepancies at the low frequency of 0.1 Hz in the horizontal component, which may cause distortion of the Cd value. Since the absolute Cd values of the W015 and the HWA032 in the east-west component are similar, a possible installation problem could also explain the discrepancy. Additional investigations at this station site are needed. 

With data from the dense, real-time *P*-Alert seismic network, we can provide near-real-time Cd (>2 cm) information surrounding the epicentral location of an earthquake (M_L_ > 6) to decision-makers for the purpose of emergency response. Ideally, a finite-fault model, which is a crucial component in seismic hazard mitigation, could be obtained directly from the Cd values derived through the *P*-Alert data. The *P*-Alert network, which is based on low-cost MEMS accelerometers, can be easily implemented in other places around the world for research on EEW systems, structural health monitoring, near-real-time shaking maps and Cd value.

## Figures and Tables

**Table 1:** Results of coseismic deformation (Cd) derived from the *P*-Alert, the TSMIP, and the GPS. The word “none” indicates Cd values less than 2 cm. The two superscript numbers indicate the building floor of instrument location (left) and the total number of stories of building (right), respectively. For example, “1st, 2” indicates that an instrument was installed on 1st floor of the two-story building.

Station	U-D/N-S/E-W (cm)	SDV of U-D/N-S/E-W (cm)
W079(*P*-Alert) ^2nd,2^ TCU084(TSMIP) 0034(GPS)	+3.18/+2.34/−3.06 +5.04/+2.19/−2.34 +6.61/+3.12/−2.98	0.81/0.29/0.31 0.63/0.39/0.46 0.68/0.21/0.28
W07C(*P*-Alert) ^2nd,2^ TCU148(TSMIP)	+2.71/+3.05/none +2.13/+3.37/none	0.67/1.14/none 0.33/0.27/none
W005(*P*-Alert) ^2nd,2^ DSIN(GPS)	+6.18/−16.51/none +14.47/−3.75/none	1.40/2.04/none 0.52/0.15/none
W015(*P*-Alert) ^1st,1^ HWA032(TSMIP)	+5.57/none/−3.02 +5.85/none/+2.08	0.56/none/0.60 0.56/none/0.38
W05A(*P*-Alert) ^1st,1^ GS52(GPS)	−3.36/none/−4.98 −2.16/none/−3.26	0.43/none/0.59 0.29/none/0.36
W052(*P*-Alert) ^2nd,4^ KAU068(TSMIP) GS51(GPS)	−2.34/none/−3.75 −2.81/none/−4.22 −2.93/none/−2.70	0.53/none/0.93 0.51/none/0.77 0.41/none/0.32
